# Is perioperative use of a combination of pregabalin and naproxen superior to naproxen only in reducing pain in ankle fractures? A prospective, randomized, multicenter study

**DOI:** 10.1186/s13018-024-05321-7

**Published:** 2024-12-26

**Authors:** Gi Won Choi, Kwang Hwan Park, Yeo Kwon Yoon, Jin Woo Lee, Dong Woo Shim

**Affiliations:** 1 Department of Orthopedic Surgery, Korea University Ansan Hospital, Ansan, 15355 Korea; 2 Department of Orthopedic Surgery, Yonsei University College of Medicine, Seoul, 03722 Korea

**Keywords:** Ankle fracture, Pain, Pregabalin, Naproxen, Perioperative use

## Abstract

**Purpose:**

To compare the analgesic efficacy, adverse effects, and long-term functional outcomes of perioperative naproxen alone versus naproxen with pregabalin for treating pain in ankle fractures.

**Methods:**

This study included 70 patients who underwent operative fixation of rotatory ankle fractures. Group A received naproxen 500 mg only, and Group B received naproxen 500 mg with pregabalin 75 mg 2-hour before surgery and 12 hourly for 14 days thereafter. The minimal clinically important difference of the visual analog scale (VAS) for pain was set at 1.8 out of 10. VAS for pain, opioid consumption, and any adverse effects were recorded for 3 days postoperatively. VAS for pain was checked at 2- and 6-weeks and 3- and 6-months, and functional outcomes were measured at 3- and 6-months postoperatively.

**Results:**

Sixty-three patients (33 and 30 in groups A and B, respectively) completed the 6-month follow-up. Demographic data were similar between groups. VAS for pain did not significantly differ between the groups at any timepoint up to 6 months (*P* ≥ 0.520), with 95% confidence intervals consistently within 1.8. No significant differences were observed between groups in opioid consumption and functional outcomes (*P* ≥ 0.211). In group B, dizziness at 48-hour and somnolence at 72-hour were significantly predominant (*P* ≤ 0.05).

**Conclusion:**

Our study demonstrated comparable pain reduction between two groups following operative fixation of rotatory ankle fractures. However, side effects, including dizziness and somnolence, were predominant in Group B between 48 and 72 h.

## Introduction

Ankle fractures are common injuries, with a reported incidence of approximately 187 per 100,000 individuals per year [1, 2]. Although treatment options for these fractures vary depending on the severity, surgical intervention is often required to ensure proper healing and prevent long-term complications [1]. However, pain management following surgery remains a challenge, and patients are often at high risk of insufficient pain control postoperatively [3–5].

Opioid prescription has been a common practice as a means to control severe pain following orthopedic surgeries [6, 7]. However, the prevalence of opioid abuse and dependence in the United States increased from 0.095% in 2002 to 0.24% in 2011, and it is associated with increased postoperative morbidity and mortality [8]. In 2011, > 40,000 mortalities were attributed to drug poisoning, with 41% involving opioid analgesics [9]. Additionally, patients who consume more opioids do not experience less pain or greater satisfaction with their treatment or pain management [10].

Multimodal analgesia has been introduced as an alternative for improving postoperative pain and reducing opioid consumption. Preemptive use of this is known to prevent central sensitization by central neurons and their amplified peripheral neurons in response to noxious stimuli [11]. A combination of several adjunctive agents, including non-steroidal anti-inflammatory drugs (NSAIDs), neuromodulatory agents, acetaminophen, and/or neuraxial blockades and local anesthesia, has been used [12]. Among these, the most commonly used combination is likely to include NSAIDs and a neuromodulatory agent capable of blocking both central and peripheral pain pathways. However, studies on the effectiveness of this combination in ankle surgery are lacking. This study aimed to compare the analgesic effects of perioperative NSAIDs and NSAIDs combined with pregabalin in patients undergoing rotational ankle fracture surgery. We hypothesized that a combination of NSAIDs and pregabalin would be superior in pain reduction compared to NSAIDs alone.

## Methods

This study was a prospective, randomized, single-blinded multicenter trial conducted in three hospitals involving 70 patients aged 19–65 years. Approval was obtained from our institutional review board (IS19MIME0059), and the trial was registered with the Clinical Research Information Service (CRIS, KCT0007008). Written informed consent was obtained from all the participants.

Inclusion criteria included patients with rotatory ankle fractures (unimalleolar, bimalleolar, or trimalleolar fractures) who underwent operative fixation within two weeks of trauma under general anesthesia. Exclusion criteria included patients with pilon, open, or multiple fractures other than ankle fractures, those undergoing spinal anesthesia, those not opting for patient-controlled analgesia (PCA), individuals with Charcot arthropathy, chronic renal failure, diabetes mellitus for > 5 years, a history of angina pectoris or myocardial infarction within the last year, current use of pregabalin or NSAIDs, a previous surgical history of an ipsilateral ankle, ankle osteoarthritis, inflammatory arthritis, allergies to pregabalin or NSAIDs, and pregnant or nursing mothers.

According to an excel generated block randomization in the current study, the patients were stratified into two groups. Group A received naproxen 500 mg alone, and group B received naproxen 500 mg and pregabalin 75 mg, administered 2 h before surgery and then every 12 h for 14 days. Anesthesia was induced with propofol 1.5–2.5 mg/kg, rocuronium 0.6 mg/kg, remifentanil 0.2 mcg/kg/min, and sevoflurane 2–3 vol%. Anesthesia was maintained with sevoflurane and continuous remifentanil infusion (0.2 mcg/kg/min). During surgery, the following parameters were maintained: fraction of inspired oxygen (FiO_2_), 0.5; tidal volume, 6–8 mL/ideal body weight; and positive end expiratory pressure, 5 cmH_2_O. Respiratory rate was adjusted to maintain an end-tidal carbon dioxide of 35–40 mmHg. At the end of the surgery, muscle relaxation was reversed using neostigmine 1.5 mg and glycopyrrolate 0.4 mg. After airway device removal, the patient was transferred to a post-anesthetic care unit. The intravenous (IV) PCA regimen comprised citric acid fentanyl 8 µg/kg/day for those < 65 years old or 6 µg/kg/day for those > 65 years old, 100 mL of normal saline 0.9%, and ramosetron 0.3 mg. The infusion was programmed to be administered at 2 mL/h as a background infusion, with an additional 0.5 mL bolus available per demand, subject to a 15 min lockout period. Patient-controlled analgesia was initiated immediately after the operation was completed. In case of excessive pain, patients were given IV pethidine 25 mg as a rescue drug when their visual analog scale (VAS) score exceeded 5. Pethidine usage was permitted with a minimum of 4 h intervals and a maximum of six ampules per day, under the supervision of a physician. The time to first use and the quantity of ampules used were documented if used.

The VAS scores, along with any side effects, including indigestion, heartburn, general edema, dizziness, nausea, vomiting, and somnolence, were evaluated at 6, 12, 24, 48, and 72 h postoperatively. Patients were discharged three days after the operation, and prescribed the same medication to be taken in advance until 14 days postoperatively. Routine chemistry assessments, including aspartate aminotransferase, alanine aminotransferase, blood urea nitrogen, creatinine, radiographs, VAS scores, and the aforementioned side effects, were reviewed during the outpatient clinic visit at the two weeks mark. Subsequently, the patients were followed-up at three and six months postoperatively, with assessments, including VAS scores, Olerud and Molander score (OMS), and ankle-fracture outcome of rehabilitation measure (A-FORM). The use of A-FORM was permitted by the developer’s group, and both outcome measurements were validated tools for assessing recovery after ankle fractures [13]. A physician blinded to the current study evaluated all the outcomes.

### Sample size calculation

We aimed to detect the minimally clinical important difference (MCID) between the groups in the VAS pain score at 1.8 of 10 [14]. A previous study showed that anticipated pain relief after use of those drugs were 3.55 (standard deviation [SD] = 1.36) and 2.57 (SD = 1.03), respectively [15]. We assumed a sample size of 35 patients in each group, with a 5% alpha set, 15% beta error, and 20% dropout rate using G power (version 3.1.9.4, Germany). The patients were divided into two groups based on block randomization generated in Excel, with a block size of 4.

### Statistical analysis

An intention-to-treat analysis was employed, where the last data collected for patients who dropped out from further evaluations were used in subsequent analyses. Patient characteristics and clinical outcomes were presented as mean (SD) or count (percentage). The Shapiro–Wilk normality test was initially performed to assess the normal distribution of the study variables in the two groups. Upon confirmation of the normal distributions, the Student t-test for quantitative variables and chi-square test for categorical variables were used to compare between the groups. Pearson’s correlation test was used to evaluate correlations between fracture severity and other categories. Pearson’s rho values were interpreted as follows: little ( ± < 0.3), low (± 0.3–0.5), moderate (± 0.5–0.7), high (± 0.7–0.9), and very high ( ± > 0.9) [16]. Statistical significance was set at *P* < 0.05, and all statistical analyses were performed using Statistical Package for the Social Sciences (SPSS, version 25.0, IBM Corp., Armonk, NY, USA).

## Results

**Fig. 1:**
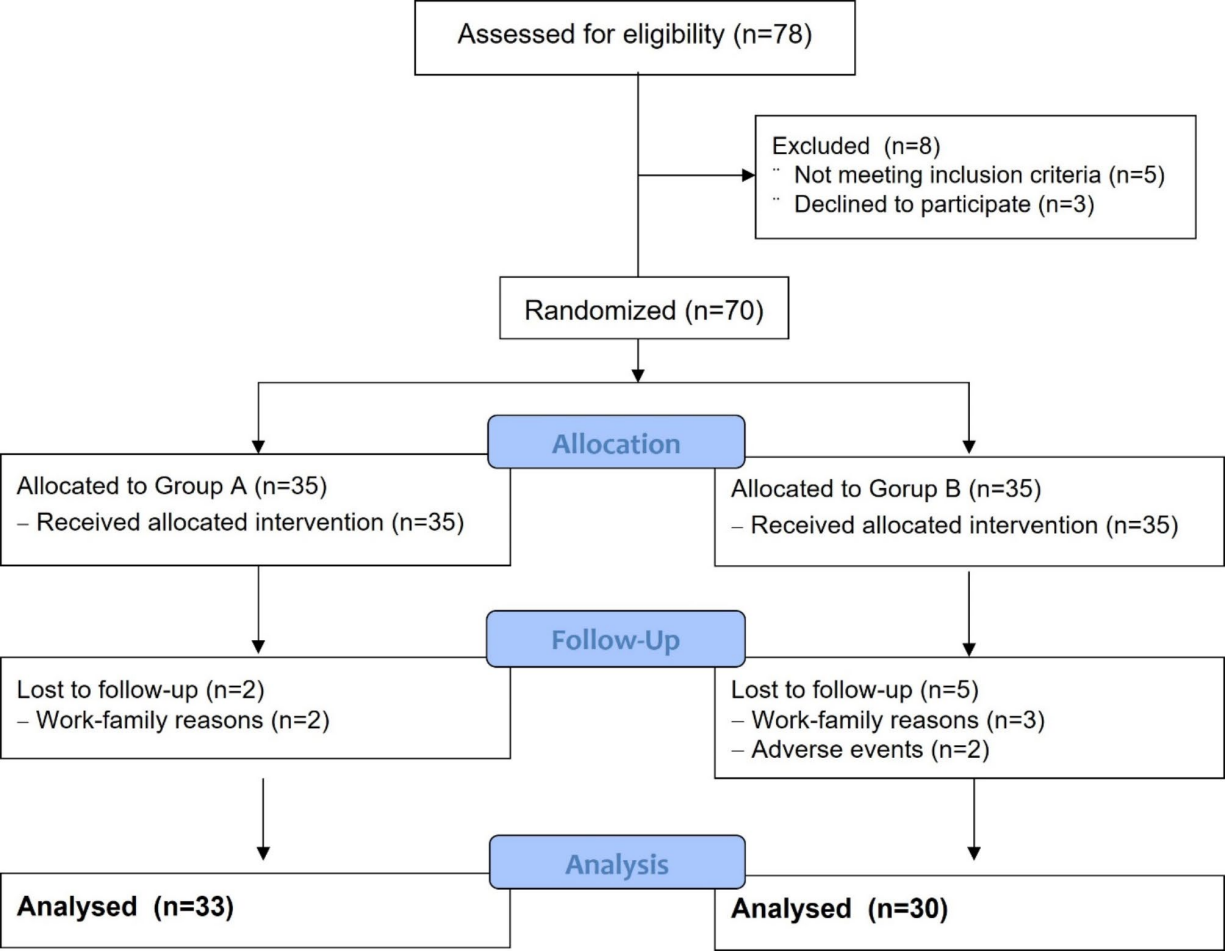
CONSORT flow diagram for the participation of study participants

The CONSORT flowchart is depicted in Fig. 1. We initially randomized 70 participants from the 78 screened between November 2019 and June 2021. At the six-month mark, 63 (90.0%) participants successfully completed their follow-up. In Group A, 2 participants dropped out of the follow-up due to work-family reasons, while in Group B, 3 participants did so for the same reason. Additionally, 2 participants from Group B withdrew from the clinical study due to symptoms of dizziness and somnolence. Demographic data, including age, sex, body mass index, injured malleoli, and past medical history, were comparable between the two groups (Table 1). There were no significant differences in time to operation, tourniquet time, use of rescue medication, and time to the first use of analgesia. In all patients, no complications such as infection, wound complications, nonunion, or implant failure were observed.

**Table 1:** Demographic data of all groups

	Group A	Group B	*P*-value
Age (year), mean (SD)	42.3 (14.7)	44.3 (13.9)	0.792
Male, N (%)	18 (54.5)	11 (39.3)	0.234
BMI (kg/m^2^), mean (SD)	25.0 (3.4)	25.1 (3.4)	0.947
HTN, N (%)	7 (21.2)	3 (10.7)	0.270
DM, N (%)	0 (0)	1 (3.4)	0.468
Injured malleoli (Uni/Bi/Tri), N	20/4/9	18/6/5	0.589
Time to operation (day), mean (SD)	4.4 (0.8)	2.4 (0.5)	0.538
Tourniquet time (min), mean (SD)	69.4 (42.3)	66.9 (40.5)	0.811
Pethidine use, N (%)	15 (45.5)	17 (58.6)	0.301
Time to escape drug (min), mean (SD)	686.9 (791.9)	789.9 (1250.0)	0.786
AST/ALT elevation, N (%)	2 (6.0)	1 (3.4)	0.676
BUN/Cr elevation, N (%)	2 (6.1)	2 (6.9)	> 0.999
Side effects, N (%)	3.9 (0.7)	4.1 (0.8)	0.279

BMI, body mass index; HTN, hypertension; DM, diabetes mellitus; AST, aspartate aminotransferase; ALT, alanine aminotransferase; BUN, blood urea nitrogen; Cr, creatinine; SD, standard deviation

The VAS scores for pain at each time point up to six months did not show significant differences, with 95% confidence intervals (CI) consistently falling within the MCID range (Table 2). Additionally, no significant differences were observed between the two groups in terms of functional outcomes, including OMS and A-FORM.

**Table 2:** Comparison of clinical outcomes between groups

	Group A	Group B	95% CI	*P* value
Initial VAS	4.6 (1.7)	4.8 (1.7)	-1.4–0.5	0.388
VAS 6 h	4.2 (2.3)	4.4 (2.7)	-1.6–0.8	0.520
VAS 12 h	3.7 (2.0)	3.1 (2.3)	-1.4–1.0	0.741
VAS 24 h	2.9 (1.7)	2.6 (2.4)	-1.1–1.0	0.901
VAS 48 h	2.7 (1.5)	2.9 (2.8)	-1.4–0.8	0.596
VAS 72 h	2.2 (1.7)	1.6 (1.7)	-0.9–1.0	0.867
VAS 2w	1.8 (1.5)	1.7 (1.7)	-0.9–0.9	0.982
VAS 6w	1.5 (1.8)	1.6 (2.0)	-1.1–0.8	0.754
VAS 3 m	1.7 (1.8)	1.6 (1.4)	-1.0–0.6	0.657
OMS 3 m	56.9 (29.0)	65.7 (24.1)	-22.7–5.1	0.211
A-FORM 3 m	55.2 (32.4)	53.5 (32.8)	-15.2–18.6	0.839
VAS 6 m	1.2 (1.8)	1.0 (1.7)	-0.7–1.3	0.546
OMS 6 m	68.7 (33.7)	70.2 (31.5)	-21.0–18.0	0.877
A-FORM 6 m	51.8 (38.0)	66.1 (40.0)	-37.2–8.6	0.215

VAS, visual analog scale; OMS, Olerud and Molander score; A-FORM, ankle-fracture outcome of rehabilitation measure; CI, confidence interval

There was no significant difference between the two groups in the number of patients experiencing side effects (*P* = 0.279). However, side effects, such as dizziness at 48 h and somnolence at 72 h were significantly more apparent in group B (*P* = 0.044 each, respectively) (Table 3). Moreover, somnolence at 48 h tended to be more prevalent in group B (*P* = 0.068).

**Table 3:** Comparison of side effects between two groups

	6 h			12 h			24 h			48 h				72 h				2w		
	A	B	*P*	A	B	*P*	A	B	*P*	A	B	*P*	A		B	*P*	A		B	*P*
Indigestion, N (%)	1 (3.0)	0	> 0.999	0	0	> 0.999	0	0	1.0	0	2 (6.9)	0.215	0		2 (6.9)	0.215	2 (6.1)		0	0.499
Heartburn, N (%)	1 (3.0)	0	> 0.999	0	0	> 0.999	0	1 (3.4)	0.468	0	0	> 0.999	0		0	> 0.999	1 (3.0)		0	> 0.999
General edema, N (%)	1 (3.0)	0	> 0.999	1 (3.0)	1 (3.4)	> 0.999	1 (3.0)	2 (6.9)	0.595	1 (3.0)	3 (10.3)	0.332	1 (3.0)		3 (10.3)	0.332	0		1 (3.4)	0.468
Dizziness, N (%)	6 (18.2)	6 (20.7)	0.803	4 (12.1)	2 (6.9)	0.676	4 (12.1)	6 (20.7)	0.360	**1 ** **(3.0)**	**6** **(20.7)**	**0.05**	3 (9.1)		2 (6.9)	0.468	1 (3.0)		1 (3.4)	> 0.999
Nausea/ Vomiting, N (%)	4 (12.1)	2 (6.9)	0.676	1 (3.0)	2 (6.9)	0.595	2 (6.1)	2 (6.9)	> 0.999	0	2 (6.9)	0.215	0		0	> 0.999	0		0	> 0.999
Somnolence, N (%)	5 (15.2)	8 (27.6)	0.230	7 (21.2)	8 (27.6)	0.559	6 (18.2)	7 (24.1)	0.565	4 (12.1)	9 (31.0)	0.068	**1** **(3.0)**		**6** **(20.7)**	**0.044**	3 (9.1)		2 (6.9)	0.468

Table 4 shows the correlation between the severity of the injured malleoli and other factors. As the number of involved malleoli increased, the operation time lengthened (moderate association), pain VAS at 6, 24, and 72 h was higher (little to low association), and functional outcomes measured using the A-FORM were significantly worse at three and six months postoperatively (low association).

**Table 4:** Correlation analysis between severity of injury and other factors

	Pearson correlation efficient (*r*’)	*P* value
Tourniquet time	**0.671**	**< 0.001**
Initial VAS	0.044	0.773
VAS 6 h	**0.383**	**0.002**
VAS 12 h	0.187	0.152
VAS 24 h	**0.363**	**0.004**
VAS 48 h	0.229	0.079
VAS 72 h	**0.283**	**0.027**
VAS 2w	0.172	0.210
VAS 6w	-0.093	0.493
VAS 3 m	0.069	0.597
OMS 3 m	0.212	0.107
A-FORM 3 m	**-0.397**	**0.002**
VAS 6 m	-0.048	0.739
OMS 6 m	0.015	0.919
A-FORM 6 m	**-0.415**	**0.003**

VAS, visual analog scale; OMS, Olerud and Molander score; A-FORM, ankle-fracture outcome of rehabilitation measure

Pearson’s rho values were interpreted as follows: little ( ± < 0.3), low (± 0.3–0.5), moderate (± 0.5–0.7), high (± 0.7–0.9), and very high ( ± > 0.9)

## Discussion

Despite pregabalin commonly serving as an adjunctive medication for postoperative pain, our study demonstrated comparable pain reduction outcomes in the perioperative use of naproxen alone and naproxen with pregabalin for rotatory ankle fractures. However, notable side effects, including dizziness and somnolence, were more prevalent in the naproxen with pregabalin group on days 2–3 postoperatively. The severity of the injury was associated with prolonged operation time, increased short-term pain, and poorer functional outcomes.

Pain after ankle fracture surgery could be devastating unless treated properly. Rbia et al. reported 23% of persistent neuropathic pain symptoms of 271 patients, which caused an impaired health-related quality of life [17]. High levels of acute pain after total knee arthroplasty are associated with increased rates of chronic postsurgical pain, suggesting that improved treatment of acute pain may lower the risk of chronic pain [18].

Opioid prescription has been common for controlling severe pain following orthopedic surgeries. Gardner et al. showed that 82.7% of patients received a discharge opioid prescription for ankle fractures; 17% contained a strong opioid [19]. However, an opioid does not improve patient satisfaction or pain, and that pain reduction effects from other regimens should be considered if possible [20].

The preoperative administrations of NSAIDs and cyclooxygenase-2 inhibitors are known to provide analgesic effect by reducing peripheral and central sensitization effectively through inhibition of prostaglandin synthesis [21–23]. Furthermore, such preemptive medications have been shown to reduce postoperative pain intensity, thereby lowering opioid requirements and related side effects. In addition to NSAIDs, anti-neuropathic drugs given preoperatively have also been suggested to reduce postoperative pain and opioid use by reducing the occurrence of central sensitization, although this is controversial [12, 24]. NSAIDs combined with pregabalin showed superior pain reduction and functional outcomes compared to placebo or NSAIDs alone in patients who underwent total hip arthroplasty, total knee arthroplasty, posterior lumbar interbody fusion, or thoracotomy [15, 25–27]. In contrast, pregabalin did not reduce analgesic use after cosmetic or ankle surgery [28, 29]. Gabapentin did not reduce morphine consumption or pain scores, and did not improve patient satisfaction after TKA [30]. A meta-analysis found that pregabalin reduced postoperative pain and analgesic drug intake, but only at doses ≥ 300 mg daily [31].

The inconsistent results of pregabalin for ankle joint pain, as seen in the current study and elsewhere, may be attributed to the location of the pathology. Yadeau et al. first proposed the potential confusing effect of regional anesthesia for ankle surgery on blocking central pain sensitization [29]. Additionally, they emphasized a sufficient amount of pregabalin for > 3 days to reduce the duration and severity of the analgesic gap. However, in this study, we used general anesthesia and longer duration of pregabalin (14 days) than previously suggested. Sidon et al. showed that neuropathic pain after foot and ankle surgery occurred in 12.4% of 533 patients, whereas lower back pain and knee osteoarthritis occurred in 53% and 34%, respectively [32]. Further, the rate of neuropathic pain in the ankle and hindfoot region was higher than that in the midfoot and forefoot (15.5% vs. 11.4% vs. 7.5%). Although no explanation was provided, they predicted that the more proximal the location of the pathology, the greater the number nerves crossing the area, which could lead to sensitization. Similarly, Tampin et al. reported that cervical radiculopathy exhibited significantly higher pain intensities, more severe pain attacks, and evoked pain by light pressure compared with carpal tunnel syndrome [33].

Gossett et al. demonstrated that, compared to closed treatment of a distal fibula fracture, only two subtypes of surgical treatment exhibited significantly higher rates of persistent opioid use than the closed treatment group: open treatment for bimalleolar ankle fractures (adjusted odds ratio [aOR], 1.32; *P* = 0.002) and trimalleolar ankle fractures with posterior lip fixation (aOR, 1.47; *P* = 0.027) [34]. Moreover, all treatment groups for ankle fractures exhibited elevated rates of new persistent opioid use, and sustained use did not directly correlate with the severity of injury. Additionally, Segal et al. reported suboptimal clinical outcomes in bi- or tri-malleolar fractures compared to uni-malleolar fractures [35]. The findings of this study consistent with those of the aforementioned studies. As injury severity increased, acute pain also increased, and short-term functional outcomes up to six months were significantly inferior.

This study has some limitations. First, we administered only one dose of pregabalin. A previous study showed that preoperative administration of pregabalin 150 mg but not 75 mg significantly reduced opioid consumption and the use of additional rescue drug [36]. However, we opted for a daily dosage of 150 mg postoperatively, as opposed to the 75 mg given twice daily, to avoid exacerbating the observed side effects in this study. Second, we included 35 patients in each group. The sample size may have been insufficient for a robust power analysis, underscoring the need for future studies to include a wider range of drug dosages and larger patient cohorts. Despite this limitation, the study has notable strengths, particularly its prospective, blinded, and randomized design. Additionally, pregabalin was administered for a sufficiently long duration of 14 days, allowing its effects to be adequately observed. To build on these findings, future research with larger cohorts and a double-blind design is planned, aiming to validate and strengthen the conclusions drawn from this study. Additionally, the study evaluated various outcomes, including pain scores at different intervals and under different conditions, opioid intake, and analgesic side effects, and also included laboratory tests. Finally, the limited generalizability of our findings warrants careful consideration. Our study sample was drawn from a specific population of ankle fractures, which may not fully represent broader demographics. Expanding the sample to include more diverse populations in future studies will help ensure broader applicability of the results.

In conclusion, our study demonstrated comparable pain reduction between two groups following operative fixation of rotatory ankle fractures. However, side effects, including dizziness and somnolence, were predominant in Group B between 48 and 72 h.

## Data Availability

The datasets generated and analyzed during the current study are not publicly available but are available from the corresponding author on reasonable request.

## Declarations

### Clinical trial registration

This trial was registered with the Clinical Research Information Service (CRIS, KCT0007008, https://cris.nih.go.kr/cris/index/index.do).
